# UV-He-Ne Laser Sequential Mutagenesis Improves Protease Production of *Bacillus velezensis*: Comparison with Traditional UV Mutagenesis

**DOI:** 10.3390/foods15152579

**Published:** 2026-07-23

**Authors:** Yuxuan Liu, Yuqing Duan, Lin Luo, Jamila A. Tuly, Haile Ma

**Affiliations:** 1 School of Food and Biological Engineering, Jiangsu University, 301 Xuefu Road, Zhenjiang 212013, China; 2 Department of Biological Sciences, National University of Singapore, 14 Science Drive 4, Singapore 117543, Singapore; 3 Institute of Food Physical Processing, Jiangsu University, 301 Xuefu Road, Zhenjiang 212013, China; 4 Guangdong Provincial Key Laboratory of Plant Adaptation and Molecular Design, Innovation Center for Cell Signal Transduction and Synthetic Biology, School of Life Sciences, Guangzhou University, Guangzhou 510006, China

**Keywords:** *Bacillus velezensis*, multiple-light-source mutagenesis, UV mutagenesis, He-Ne laser, microbial metabolism, proteomic analysis

## Abstract

This study combined the damage effect of UV and the repair effect of He-Ne laser, employing UV-He-Ne laser (UV-LA) sequential mutagenesis to improve the protease-producing ability of *Bacillus velezensis*. Compared with UV mutagenesis, UV-LA treatment elevated the positive mutation rate from 12.50% to 16.67%, and the enzyme activity of the mutant strain increased from 20.81 U/mL to 34.04 U/mL. Following 20 passages, the degree of enzyme production decline of the UV-LA strain was reduced from 44% to 23% compared with that of the UV strain, showing better genetic stability. Subsequent investigation revealed that the physiological activity (L-LDH, ATP content) and the activities of various key metabolic enzymes (PFK, IDH, ATPase) in the UV-LA strain exceeded those in the UV strain. Genome sequencing results showed that the UV-LA strain had fewer mutations than the UV strain, and the mutation sites were more concentrated in the regulatory region. Proteomic analysis identified 108 differentially expressed proteins (56 upregulated and 52 downregulated) between the UV strain and UV-LA strain, primarily associated with bacterial energy metabolism, translation, and secretion systems. Research indicates that UV-LA treatment may leverage the laser’s light effects to reduce UV damage, thereby achieving regulation of metabolic and secretory processes. This study is expected to develop a novel and efficient mutagenesis method for microbial breeding and to offer new ideas for expanding the application of UV mutagenesis technology.

## 1. Introduction

Fermentation is a core process in contemporary biomanufacturing and is extensively utilized in food processing, feed additive production, and related fields [1]. This process boosts the nutritional content and digestibility of raw materials through biotransformation, while also contributing to the degradation of antinutritional factors and the development of distinctive product flavors [2,3]. In biological fermentation, the performance of protease-producing strains directly determines the efficiency of converting proteins into polypeptides and amino acids [4]. It is also a key factor affecting the fermentation cycle, product quality, and economic benefits [5]. *B. velezensis* is a commonly used fermentative microorganism with strong protease secretion capacity [6,7,8]. However, wild-type strains generally exhibit inherent limitations, including low metabolite production, intricate by-product compositions, and poor environmental resilience [9]. Consequently, advancements are required to satisfy the demands of industrial fermentation production for elevated yield and high stability [10]. Nevertheless, microbial enzyme production is governed by complex metabolic regulation, making targeted engineering to enhance enzyme productivity extremely challenging. In contrast, mutation breeding, owing to its randomness and broad applicability, has been widely employed to improve microbial strain performance [5,11].

Compared with chemical mutagenesis, which relies on chemical mutagens, physical mutagenesis directly acts on genetic material via physical energy [12]. It offers several advantages, including the absence of chemical residues, a broader mutation spectrum, and greater operational control. Among these approaches, ultraviolet (UV) mutagenesis is one of the most prevalent physical mutagenesis methods. It mainly induces the formation of covalently linked pyrimidine dimers between adjacent pyrimidine bases, thereby interfering with DNA replication and transcription and ultimately altering genetic information [13]. However, UV mutagenesis primarily exerts a single damaging effect and usually requires a relatively high lethal rate to achieve satisfactory mutagenic efficiency, potentially imposing excessive damage on the strain [10]. This causes the mutant strain to experience a decline in performance or loss of high-yield traits during continuous subculture or fermentation [14]. In addition, repeated use of a single physical mutagen may lead to the development of strain tolerance, particularly during continuous mutagenesis, thereby reducing mutation efficiency [15]. Accordingly, compound mutagenesis strategies have attracted increasing attention. By integrating different mutagenic factors, these strategies aim to improve mutagenic efficiency through synergistic and cumulative effects while expanding the screening space for desirable mutants. In recent years, the biological effects of He-Ne laser irradiation have received growing attention [16]. Unlike UV treatment, low-dose or short-duration He-Ne laser exposure can transiently enhance microbial respiration and ATP synthesis. In addition, our previous research found that He-Ne laser may promote the expression of bacterial repair systems [17]. Therefore, combining the damaging effects of UV with the repair-promoting effects of the He-Ne laser may provide a new strategy to overcome the limitations associated with single-UV mutagenesis.

This study innovatively incorporated the biological effects of UV and He-Ne laser. A sequential treatment strategy involving UV followed by He-Ne laser irradiation (UV-LA) was designed and implemented to screen for high-protease-producing *B. velezensis*. Unlike our previous research, the present study focused on the impact of the He-Ne laser repair’s effect on the conventional UV mutagenesis process and designed corresponding comparative experiments around this core scientific issue [10,17]. Initially, from a methodological perspective, alterations in strain lethality were analyzed to verify the existence of damage and repair effects during the sequential treatment process. Subsequently, high-throughput screening was employed to identify the optimal mutant strains generated under UV and UV-LA treatment modes, respectively. By comparing the positive mutation rate, protease activity, cell viability, the activities of key metabolic enzymes, and indices related to substance transport, together with proteomic analysis, the potential advantages of this compound mutagenesis strategy were systematically elucidated from the perspective of metabolic regulation. This study is expected to provide a novel and efficient mutagenesis strategy for microbial breeding and to establish a theoretical basis for the application of compound physical mutagenesis in industrial microbial breeding.

## 2. Materials and Methods

### 2.1. Materials

*Bacillus velezensis* (CICC 23571) was purchased from the China Center of Industrial Culture Collection (Beijing, China). FITC-casein was purchased from Thermo Fisher Scientific (Waltham, MA, USA). L-Lactate Dehydrogenase activity assay kit (BC0685), luminescent microbial cell viability assay kit (CA3700), Phosphofructokinase activity detection kit (BC0530), Isocitrate dehydrogenase activity detection kit (BC0400), and ATPase activity kit (BC0960, BC0060) were purchased from Solarbio Science & Technology Co., Ltd. (Beijing, China). All other reagents were of analytical grade and purchased from Sinopharm Chemical Reagent Co., Ltd. (Beijing, China).

### 2.2. Determination of Growth Curve

According to the method described by Feng et al. [18], the activated strain was inoculated into 100 mL of LB liquid medium and incubated at 30 °C for 36 h. During the incubation period, the absorbance of the culture at 600 nm was measured. The growth curve was then illustrated based on incubation time and OD_600_ values.

### 2.3. Microbial Mutagenesis

The strain was mutagenized using multi-light-source mutagenesis equipment (MLME, manufactured by Jiangsu University, Zhenjiang, China). The device is equipped with a UV light source (254 nm; Philips, The Netherlands) and a He-Ne laser source (632 nm; Melles Griot Inc., Carlsbad, CA, USA) [10,18]. The distance between the light source and the sample surface was fixed at 10 cm. The UV irradiation intensity was 1.5 mW/cm^2^, with an estimated absorption rate of approximately 99.38% in the bacterial suspension. The He-Ne laser exhibited an actual output power of 10.3 mW and a beam diameter of 0.65 mm, corresponding to a power density of approximately 3 W/cm^2^. The estimated absorption rate of laser irradiation in the bacterial suspension was approximately 99.43%.

A 5 mL aliquot of bacterial culture in mid-log phase was transferred to a sterile tube, washed twice with 3 mL of sterile saline, and resuspended. Subsequently, 100 μL of the bacterial suspension (about 5 × 10^8^ CFU/mL) was transferred to a 1.5 mL sterile tube and placed in the MLME for mutagenic treatment. The light source will illuminate the bacterial suspension from above the sample tube for maximum irradiation [2,18,19]. The sterile tube was kept open throughout the irradiation process to prevent the tube lid from blocking the incident light. The UV-treated time was set to 0–30 min, while the He-Ne laser-treated time was 0–120 min. For the sequential UV-He-Ne laser (UV-LA) treatment, the He-Ne laser treatment time was fixed at 20 min, and the UV treatment time ranged from 0 to 30 min.

The treated bacterial suspension was serially diluted and spread onto LB plates. After incubation at 30 °C for 24 h, the colonies were counted. The lethal rate was calculated using the following formula (if the calculated lethal rate was negative, this indicated that the treatment promoted bacterial growth; in that case, the absolute value was taken and recorded as the growth rate) [10]:

(1) $$L=\frac{C_{C}-C_{T}}{C_{C}}\times 100\%$$

where *L* is the lethal rate, *C*_C_ is the number of colonies in the untreated sample, and *C*_T_ is the number of colonies in the treated sample.

### 2.4. High-Throughput Screening of Protease-Producing Mutant Strains

**Figure 1:**
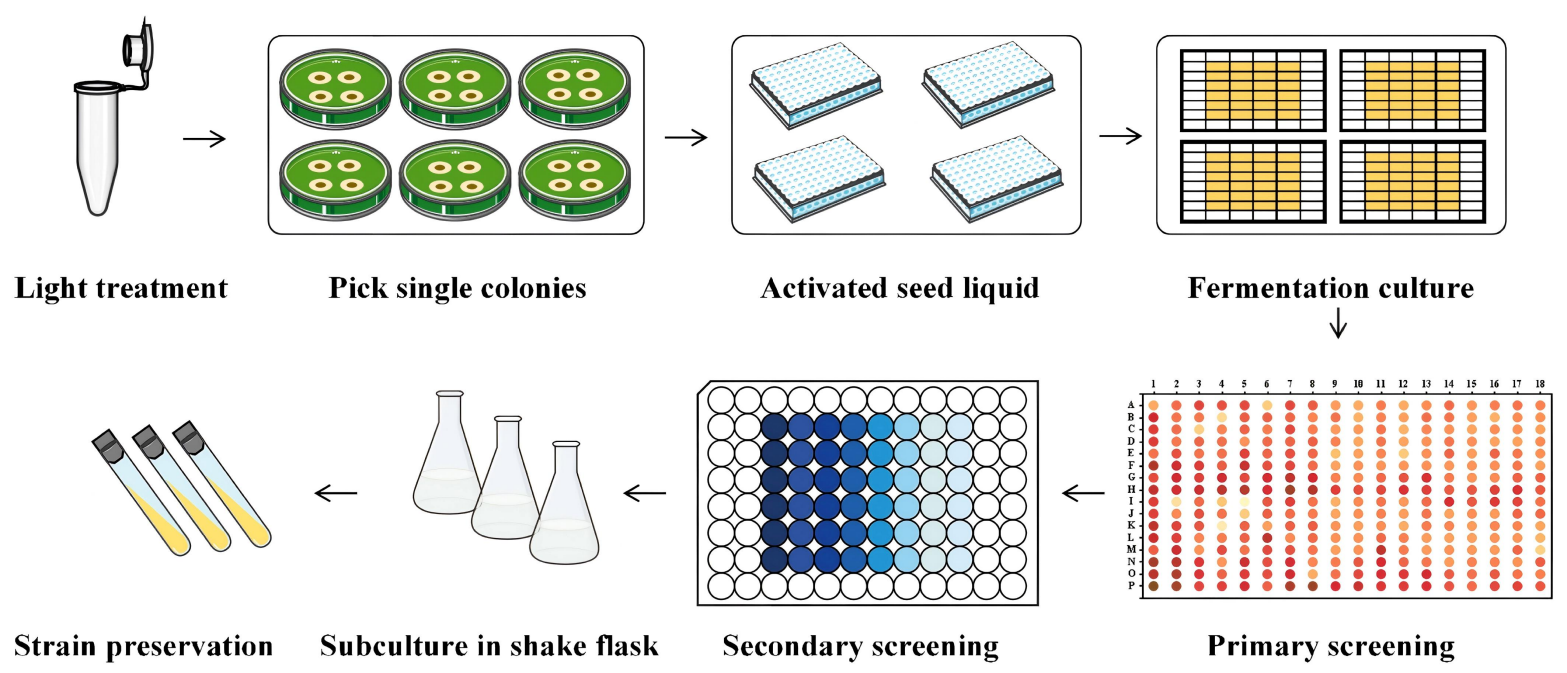
Schematic diagram of mutagenesis screening process.

A high-throughput screening method for protease-producing strains was designed according to our previous study (Figure 1) [10]. Briefly, the mutated bacterial solution was first spread onto casein plates (casein, 5 g/L; yeast extract, 5 g/L; sodium chloride, 5 g/L; disodium hydrogen phosphate dodecahydrate, 2 g/L; bromothymol blue, 0.05 g/L; agar, 15 g/L). After incubating at 30 °C for 24 h, single colonies exhibiting larger hydrolysis zones were selected and cultured in a 96-well deep-well plate with shaking. The resulting cultures were then incubated in a 48-well deep-well plate containing 900 μL of sterile LB medium at an inoculation volume of 10% and cultured for 24 h at 30 °C and 300 r/min. Preliminary screening was performed using a fluorescent protease activity assay. The top 96 strains with the highest enzyme activity obtained from the initial screening were re-cultivated and re-screened using a UV protease activity detection method. Fluorescence and UV absorbance measurements were performed using a multifunctional microplate reader (SPARK, Tecan, Männedorf, Switzerland). Finally, the UV strain and the UV-LA strain with the highest protease activity were screened.

#### 2.4.1. Determination of Protease Activity by Fluorescence Method

Based on the experimental method described by Stonoha-Arther et al. [20], with appropriate modifications, the cultured bacterial suspension was centrifuged at 2000 rpm for 15 min. The supernatant was then collected for analysis. The experiment was conducted using a 384-well plate. Initially, 37.5 μL of the enzyme solution to be tested was added to each well, followed by incubation at 37 °C for 5 min to equilibrate. Subsequently, 37.5 μL of a 10 μg/mL FITC-casein solution was introduced into each well, and the plate was briefly centrifuged using a microplate centrifuge to ensure thorough mixing of all components. The 384-well plate must be incubated at 37 °C for 30 min. After the reaction is complete, the 384-well plate must be placed in a microplate reader, and the excitation wavelength must be set to 485 nm, and the emission wavelength to 538 nm, for fluorescence detection.

#### 2.4.2. Determination of Protease Activity by UV Method

In accordance with the method described by Lu et al. [10], the bacterial culture was subjected to centrifugation at 2000 r/min for 15 min. A total of 50 μL of supernatant was transferred to a 96-well plate and prewarmed at 30 °C for 2 min. We added 50 μL of casein solution, shook it to mix the solution, and then incubated the solution at 30 °C for 10 min. A total of 100 μL of trichloroacetic acid was added to stop the reaction, and the solution was centrifuged at 4000 rpm for 5 min. Then, 20 μL of the supernatant was transferred to a new microplate, and 100 μL of sodium carbonate solution was added, followed by 20 μL of Folin’s solution. The solution was mixed well and incubated at 30 °C for 20 min to color it. The absorbance was measured at 680 nm using a microplate reader (SPARK, Tecan, Männedorf, Switzerland). For the control group, 100 μL of trichloroacetic acid was first added to 50 μL of the test solution; then, 50 μL of casein solution was added; the remaining steps are the same.

### 2.5. Genetic Stability Test

The wild strain and mutant strains were cultured through serial passages in LB medium. Each strain was passaged 20 times, and strains from the 1st, 3rd, 5th, 10th, 15th, and 20th generations were selected for analysis. Protease activity was measured as described in Section 2.4.2 to determine the genetic stability of each mutant strain.

### 2.6. Determination of L-Lactate Dehydrogenase (L-LDH) Activity

Separate cultures of the wild strain and the mutant strain were inoculated into 10 mL of LB liquid medium and incubated with shaking at 30 °C and 180 rpm for 24 h to serve as seed cultures. Using a 1% inoculum volume, the seed culture was transferred into 50 mL sterile shaking flasks containing 10 mL of LB medium and incubated under the same conditions for 9 h. Samples were collected during the incubation period and analyzed according to the L-LDH activity assay kit instructions (Solarbio Science & Technology Co., Ltd., Beijing, China).

### 2.7. Determination of Microbial Viability

The strains were cultured according to the method described in Section 2.6. Samples were collected during the incubation period and analyzed according to the instructions of the luminescent microbial cell viability assay kit (Solarbio Science & Technology Co., Ltd., Beijing, China).

### 2.8. Determination of Phosphofructokinase (PFK) Activity

The strains were cultured according to the method described in Section 2.6. Samples were obtained at 9 h and 20 h of incubation, respectively. A total of 5 mL of culture medium was transferred to a sterile 10 mL centrifuge tube and centrifuged at 4 °C and 8000 rpm for 10 min to obtain a bacterial pellet. One must lysis the bacterial pellet according to the instructions for the PFK activity assay kit (Solarbio Science & Technology Co., Ltd., Beijing, China) and collect the supernatant for enzyme activity measurement.

### 2.9. Determination of Isocitrate Dehydrogenase (IDH) Activity

Bacterial culture samples were collected at 9 h and 20 h of cultivation. Subsequently, 5 mL of the culture was centrifuged at 4 °C and 8000 r/min for 10 min to obtain the cell pellet. The pellet was resuspended in 400 μL of extraction buffer containing 5 mM of MnSO_4_, 2 mM of DTT, 20 mM of KCl, 0.1 mM of EDTA, and 0.1 M of Tris-HCl, and the cells were disrupted using a tissue grinder (SCIENTZ-48L, Scientz Biotechnology Co., Ltd., Ningbo, China) at 30 Hz for 10 min at 4 °C. The disrupted mixture was then centrifuged at 4 °C and 8000 r/min for 10 min, and the enzyme activity in the supernatant was determined according to the instructions of the IDH activity assay kit (Solarbio Science & Technology Co., Ltd., Beijing, China).

### 2.10. Determination of ATPase Activity

Based on the method of Hao et al. [21], with some modifications, determination of the ATPase activity was carried out as follows. A 1 mL aliquot of seed culture was inoculated into a 250 mL flask containing 100 mL of LB broth and incubated at 30 °C with shaking. Samples were collected at 9 and 20 h of cultivation. For enzyme extraction, 5 mL of culture was harvested by centrifugation at 8000 r/min for 10 min at 4 °C, and the supernatant was discarded. The cell pellet was resuspended in 1 mL of lysis buffer and then disrupted by ultrasonication in an ice bath at 20 kHz for 10 min, with a pulse cycle of 3 s on and 10 s off. The lysate was then centrifuged at 10,000 r/min for 10 min at 4 °C, and the supernatant was collected for analysis. The activities of Ca^2+^/Mg^2+^-ATPase and Na^+^/K^+^-ATPase were determined using commercial assay kits (Solarbio Science & Technology Co., Ltd., Beijing, China) according to the instructions.

### 2.11. Whole-Genome Resequencing

The wild-type strain and the two mutant strains were individually inoculated into 10 mL of LB broth and cultured at 30 °C with shaking at 180 r/min for 12 h. The cultures were then centrifuged at 4 °C and 8000 r/min for 10 min. After removal of the supernatant, the cell pellets were washed twice with sterile physiological saline. During the final washing step, the cells were transferred into sterile 1.5 mL microcentrifuge tubes. Following an additional centrifugation step, the cell pellets were rapidly frozen in liquid nitrogen for 1 min and subsequently stored at −80 °C. Whole-genome resequencing was performed by Sangon Biotech Co., Ltd. (Shanghai, China).

### 2.12. Proteomics Assays

Bacterial cells cultured for 24 h were collected by centrifugation at 10,000 r/min for 8 min at 4 °C, washed with sterile saline, flash-frozen in liquid nitrogen, and stored at −80 °C. Proteins were extracted and digested using the iST sample preparation kit (PreOmics, Martinsried, Germany). The resulting peptides were desalted, vacuum-dried, and reconstituted in 0.1% formic acid containing iRT peptides (Biognosys AG, Schlieren, Switzerland). Peptides (200 ng) were separated on an UltiMate 3000 system (Thermo Fisher Scientific, MA, USA) equipped with an AUR3-15075 C18 column (15 cm × 75 μm, 1.7 μm; IonOpticks Pty Ltd., Collingwood, Australia) at 50 °C and 400 nL/min. The gradient of mobile phase B (80% acetonitrile, 0.1% formic acid) was 4–28% over 25 min, 28–44% over 10 min, 44–90% over 10 min, held at 90% for 7 min, and re-equilibrated at 4% for 8 min. MS analysis was performed on a timsTOF Pro 2 mass spectrometer (Bruker Daltonics, Bremen, Germany) in diaPASEF mode as previously described by Meier et al. [22], with a scan range of 349–1229 m/z and an isolation window of 40 Da. Raw data were quality-checked using QuiC and analyzed with Spectronaut 14 (Biognosys AG, Schlieren, Switzerland).

### 2.13. Statistical Analysis

All experiments were performed in triplicate, and the results are expressed as mean ± standard deviation. One-way analysis of variance (ANOVA) followed by Duncan’s test (*p* < 0.05) was performed using SPSS 27.0 software (IBM Corp., Armonk, NY, USA). Graphs were drawn using OriginPro 2025 software (OriginLab Corp., Northampton, MA, USA).

## 3. Results and Discussion

### 3.1. Growth Curve Analysis

**Figure 2:**
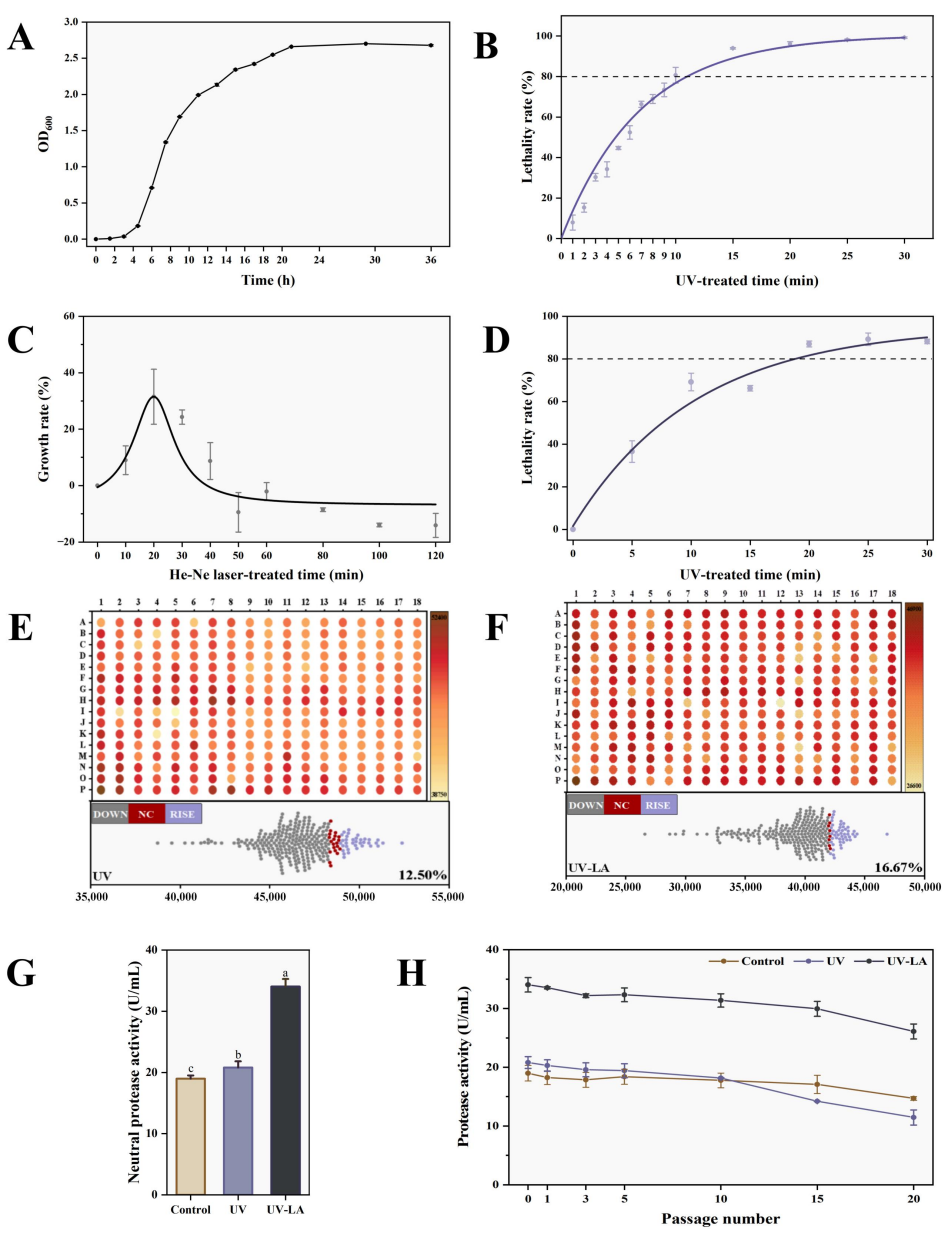
(**A**) Growth curve of *Bacillus velezensis*. (**B**) Effect of UV treatment on the lethality of *Bacillus velezensis*. (**C**) Effect of He-Ne laser treatment on the growth rate of *Bacillus velezensis*. (**D**) Effect of UV-LA sequential treatment on the lethality of *Bacillus velezensis*. (**E**) Enzyme activity distribution and positive mutation rate statistics of preliminary screening by UV mutagenesis. (**F**) Enzyme activity distribution and positive mutation rate statistics of preliminary screening by UV-LA mutagenesis. (**G**) Protease activity of each model mutant strain. (**H**) Effect of passage number on strain protease activity. Different lowercase letters indicate significant differences among groups (*p* < 0.05).

The physiological state of bacterial strains varies across different growth stages. Therefore, understanding their growth patterns is crucial for subsequent mutagenesis. Figure 2A illustrates the growth process of *B. velezensis*, which primarily exhibits four distinct phases. During the initial 0–4 h, the strain enters a lag phase, characterized by slow cell proliferation and no significant change in OD_600_. During this period, the strain progressively adapts to the new growth environment, adjusts its metabolism, and accumulates the necessary substances for subsequent growth [23]. From 4 to 18 h, the strain enters the log phase, during which cells proliferate rapidly, and cell numbers increase exponentially. Throughout this stage, the cells exhibit a relatively uniform physiological state and heightened sensitivity to external stimuli, making it an optimal time for mutagenesis treatment [18]. Between 18 and 29 h, the strain enters the stationary phase, during which the growth rate slows significantly, and the cell count remains stable. At this point, nutrients in the culture medium are gradually depleted, and metabolic products accumulate continuously. When the cultivation time exceeds 29 h, the strain enters the decline phase, during which bacterial mortality increases. Based on the characteristics of each growth phase, cells cultured for 12 h were selected for subsequent mutagenesis experiments.

### 3.2. Changes in Lethality Under Different Light Treatment Conditions

First, the wild strains were subjected to UV and He-Ne laser treatment, respectively, to determine the appropriate treatment time range. Figure 2B shows the effect of UV irradiation time on strain lethality. The results indicate that as irradiation time increased, lethality increased significantly, consistent with the study by Lu et al. [10]. This is because the irradiation time is positively correlated with the radiation dose received by the strain. When radiation-induced damage surpasses the physiological tolerance threshold and the repair capacity of the bacteria, the cells encounter difficulties in sustaining normal metabolic functions, ultimately leading to cell death [24]. In classical microbial mutagenesis, a lethality rate of approximately 70–90% is generally considered suitable for mutant screening because it provides a balance between mutation induction and cell survival [18,25]. Although increasing irradiation dose may enhance mutation frequency, excessive DNA damage can reduce mutant recovery efficiency and increase the occurrence of deleterious mutations. Therefore, a lethality rate close to 80% is often adopted as a practical criterion for industrial strain improvement. Based on this criterion, a UV treatment time of 10 min was selected for subsequent experiments.

Figure 2C shows the effect of the He-Ne laser on the strain. He-Ne laser treatment did not show any significant lethal effects. On the contrary, it showed a bidirectional biological effect: short-term treatment promoted the strain’s growth, whereas long-term treatment inhibited its growth or caused its death. After 20 min of irradiation, the bacterial population increased by 31.48%. It is generally accepted that the primary mechanisms by which He-Ne lasers promote microbial growth are the enhancement in ATP synthesis and the stimulation of intracellular physiological repair processes [26]. In view of the unique growth-promoting effect of the He-Ne laser, which may increase the diversity of mutagenesis results, a 20 min treatment was selected as the final laser time.

On this basis, UV-LA treatment was performed (Figure 2D). The He-Ne laser treatment time was fixed at 20 min, while the lethality rate was modulated by varying the UV treatment time. The results showed that the UV-induced lethal process was effectively delayed by the repair effect of the He-Ne laser. In single-UV treatment, the lethality rate reached 80% after 10 min of exposure. However, in the UV-LA treatment mode, the UV treatment time required to achieve 80% lethality was 20 min. This indicates that the He–Ne laser played a significant role in repairing UV-induced damage, thereby partially mitigating the lethal effects of UV on the bacterial strain. To facilitate the subsequent comparison of the two mutagenesis methods, the processing parameters of the two methods were determined based on the 80% lethality rate. Therefore, UV treatment for 20 min, followed by He-Ne laser treatment for 20 min, was used to construct a “Damage-Repair” mode to mutagenize the wild strain. The differences between this model and traditional single-UV mutagenesis for obtaining mutant strains were also discussed.

### 3.3. Mutant Strains Screening and Genetic Stability Analysis

The mutant strains produced by the two mutagenesis modes were initially screened, and 288 mutants were screened for each treatment method. The positive mutation rate was calculated based on the protease activity. The results showed that the positive mutation rate for UV mutagenesis was 12.50% (Figure 2E), and for UV-LA mutagenesis, it was 16.67% (Figure 2F). The mutation rate of UV-LA mutagenesis increased. The He-Ne laser is hypothesized to activate strain repair pathways, increase the expression efficiency, and facilitate the repair of UV-induced damage, thereby increasing the probability of obtaining positive mutant strains [17]. In addition, according to a report by Bannenberg et al. [27], the strain’s repair mechanism will preferentially act on the metabolic pathways that maintain its basic life activities, which helps to reduce the negative interference of mutagenesis on enzyme-producing metabolic pathways. From the perspective of mutation rate, UV, as a potent mutagenic light source, can provide a more extensive mutation library, while the He-Ne laser promotes the recovery and growth of individuals experiencing minimal metabolic damage. This approach also preserves the mutation characteristics of the enzyme-producing pathway, thereby enriching forward mutations in the pathway [28].

The mutants with higher enzyme activity identified in the initial screening were re-screened, and the strains with the greatest improvement in protease activity were selected. Figure 2G shows the enzyme activity of the best mutants under each treatment mode. The UV-LA strain has the highest protease activity, reaching 34.04 U/mL, approximately 79% higher than that of the wild strain, whereas the UV strain showed an increase of only about 10%. This shows that under the same screening pressure, the UV-LA mode not only has a high positive mutation rate but is also more likely to produce mutant strains with higher protease production. Based on previous experiments, it is speculated that this may be due to the strain initiating the repair system after UV damage. In particular, activating the error-prone repair system increases the likelihood of base mismatches, thereby significantly altering the bacterial phenotype [29].

To further assess the effect of He-Ne laser-induced repair on strain stability during continuous passage, the mutant strain and the wild strain were continuously subcultured, and changes in protease activity were monitored over time. The results are shown in Figure 2H. The wild strain showed the best passage stability, and its enzyme activity only decreased by about 19% after 20 generations. The decrease in its enzyme activity may be related to the gradual accumulation of spontaneous mutations during continuous passage, which tend to have negative effects, leading to a decline in strain performance [30]. In contrast, both mutant strains maintained good stability for the first five generations, but protease activity gradually decreased thereafter. This may be due to the genetic instability of the mutant strain caused by high-intensity mutagenesis. Although it acquired favorable mutations related to enzyme production, it also accumulated many recessive non-targeted mutations. As it is continuously passed in an environment lacking selective pressure, these non-targeted mutations may gradually affect the expression efficiency of key enzyme-producing genes or regulatory systems, leading to the degradation of the strain [31]. After 20 generations, the enzyme activity of the UV strain decreased by approximately 44%, whereas that of the UV-LA strain decreased by only 23%. These results indicate that UV-LA treatment enhances the stability of the mutant strain, reduces cellular damage, and slows the decline in enzyme activity.

### 3.4. L-LDH Activity Analysis

**Figure 3:**
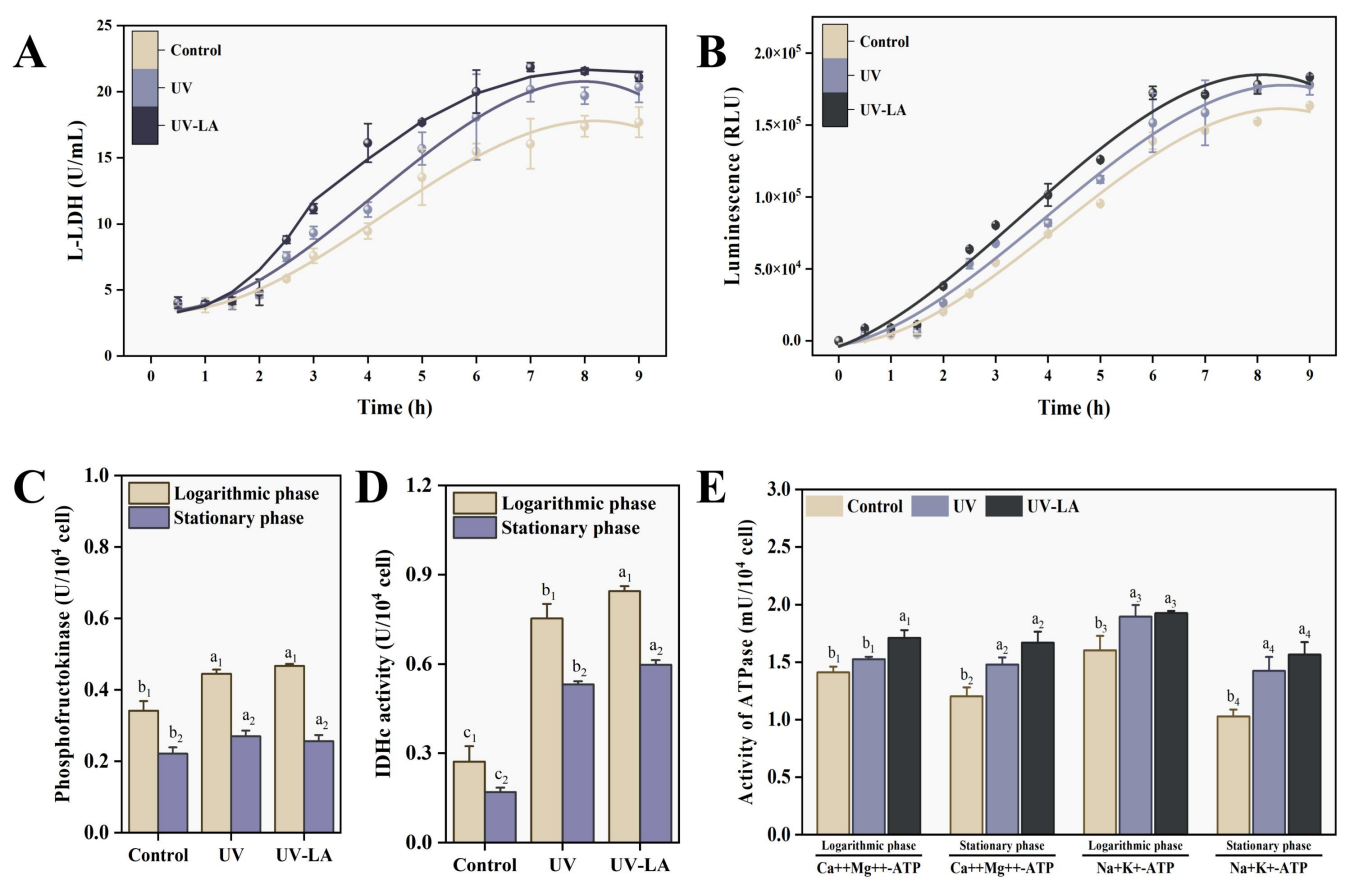
(**A**) Changes in L-LDH activity between the starting strain and the mutant strains. (**B**) Changes in total cellular ATP content of starting strain and mutant strains. (**C**) Changes in PFK activity of the starting strain and two mutant strains at different culture stages. (**D**) Changes in IDH activity of the starting strain and two mutant strains at different culture stages. (**E**) Changes in ATPase activity of starting strain and two mutant strains at different culture stages. Different lowercase letters indicate significant differences among groups (*p* < 0.05). Numerical subscripts indicate the comparison groups.

L-LDH is a crucial terminal oxidoreductase in the glycolysis pathway and plays a vital role in sustaining the basal metabolism of microorganisms [32]. Figure 3A illustrates the trend in L-LDH activity for the three strains over different culture durations. As culture duration extends, L-LDH activity gradually increases and stabilizes during the mid-logarithmic growth stage. This phenomenon is primarily associated with the increased metabolic demands of bacteria during the logarithmic growth phase. When bacteria proliferate in the logarithmic phase, it is necessary to increase the intracellular NAD^+^/NADH cycle regeneration rate by increasing enzyme activity to maintain the continuous and efficient operation of the glycolysis pathway, thus continuously providing ATP and metabolic intermediates required for cell growth [32]. During the mid-to-late logarithmic phase, the strain’s growth rate slows due to nutrient depletion and the accumulation of metabolic by-products, resulting in a plateau in enzyme activity [33]. Furthermore, the acid produced by the bacteria during the culture process will decrease the medium’s pH, potentially exerting an inhibitory influence on L-LDH activity [34]. Compared with the wild strain, the L-LDH activity of the two mutant strains was elevated. The L-LDH activity of the UV strain and UV-LA strain in the logarithmic phase increased by approximately 26% and 35%, respectively. The data demonstrate that the mutant strain exhibits enhanced metabolic flux and physiological activity, enabling it to accumulate more intermediate metabolites per unit time, hence providing materials and energy reserves for subsequent enzyme synthesis. Notably, compared with the UV strain, the UV-LA strain showed a shorter lag phase and higher L-LDH enzyme activity (increased by about 9%). These results suggest that the He-Ne laser may partially alleviate UV-induced damage, shortening the adaptation phase of the mutant strain through its repair effect and enabling it to enter a metabolically active state earlier. Leena et al. [35] also found that damaged bacteria showed a longer growth retardation period.

### 3.5. Microbial Cell Viability Analysis

ATP is an important energy carrier, and its content is significantly positively correlated with the number of viable bacteria, indicating that it serves as an important indicator of the metabolic state of microorganisms [36]. Figure 3B depicts the changes in the total intracellular ATP content of the strains at different times of culture. The results showed that the ATP content increased significantly with the prolongation of culture time, indicating that during the rapid growth stage, the ATP content was positively correlated with the strain’s growth rate and metabolic activity, which was consistent with the report of Mu et al. [37]. As the culture time approaches the late logarithmic growth phase, the ATP content begins to stabilize. At this juncture, the production and consumption of intracellular ATP attain a dynamic equilibrium, prompting the bacteria to allocate increased energy toward metabolite synthesis [38]. Moreover, according to Harwood et al. [39], ATP is used not only for energy supply to microorganisms but also as a cofactor for a variety of enzymes that regulate metabolism. Therefore, as the level of bacterial metabolism increases, ATP will be further consumed to support the synthesis and metabolic regulation of related enzyme systems [39]. Further comparisons revealed that the ATP content of both mutant strains was significantly higher than that of the wild strain at the same culture time points. The ATP content of the UV strain and UV-LA strain in the logarithmic phase increased by approximately 8% and 17%, respectively. This shows that the mutagenesis treatment significantly enhanced the oxidative phosphorylation level and substrate-level phosphorylation efficiency of the mutant strain, conferring significant advantages in cell viability and material conversion rate [38]. It is worth noting that, compared to the UV strain, the UV-LA strain exhibited faster rates of ATP synthesis and accumulation (increased by about 9%). This implies that the UV-LA strain has a higher energy load during the logarithmic growth phase and can enter the energy allocation phase earlier to support protein synthesis and secretion [39]. These results further prove that UV-LA treatment can effectively repair some non-lethal metabolic system damage caused by UV irradiation while maintaining the diversity of gene mutations. This ensures the integrity of the strain’s energy conversion system, thereby shortening the metabolic lag phase and enhancing enzyme production efficiency.

### 3.6. PFK Activity Analysis

PFK is a key rate-limiting enzyme in the glycolysis pathway and a core hub in regulating microbial energy metabolism flux [40]. Figure 3C demonstrates the changes in PFK activity of each strain under different culture stages. The results showed that PFK activity was high in all strains during the logarithmic growth phase. This may be due to the microorganisms’ increased demand for ATP during rapid growth to support cell division and cytoskeletal synthesis, with higher PFK activity ensuring an efficient energy supply. This finding is consistent with previously observed patterns of intracellular ATP levels [41]. During the stationary phase of bacterial growth, the physiological metabolism of the strain stabilizes, leading to a marked reduction in the energy requirements for growth and division, which therefore results in a substantial decrease in enzyme activity compared to the logarithmic growth phase [42]. Notably, the PFK activity of the two mutant strains was significantly higher than that of the control group (the UV strain increased by 30% and the UV-LA strain increased by 37%), indicating that mutagenesis significantly enhanced the basal metabolism and physiological vitality of the strains. However, the UV strain and the UV-LA strain did not show significant differences in PFK enzyme activity. The reason may be related to the negative feedback regulatory mechanism of ATP on PFK [43]. When the intracellular ATP content reaches a certain level, ATP can bind to PFK and induce a conformational change, thereby reducing the activity of the enzyme [43,44]. This negative feedback regulation mechanism helps prevent excessive accumulation of glycolysis products and maintain intracellular metabolic homeostasis.

### 3.7. IDH Activity Analysis

IDH is the key rate-limiting enzyme of the tricarboxylic acid cycle (TCA). An increase in its activity reflects the acceleration of the TCA cycle, indicating enhanced bacterial energy metabolism and precursor synthesis capacity [45,46]. Figure 3D shows the changes in IDH activity of the strains at different culture stages. The results indicate that IDH activity in each strain was significantly higher during the logarithmic growth phase than in the stationary phase, reflecting the greater energy metabolism demands of bacteria during rapid growth. This trend is generally consistent with the observed changes in PFK activity. The IDH activity of the mutant strain at each growth stage was markedly superior to that of the original strain, demonstrating that the mutagenesis treatment substantially enhanced the strain’s central carbon metabolism, hence enhancing substrate conversion efficiency and physiological activity [47]. Liu et al. [45] also reported that increased IDH activity can facilitate the secretion of secondary metabolites by *B. licheniformis*. This can also explain the increased protease activity of the mutant strain in this study. It is worth noting that the UV-LA strain showed higher IDH activity than other strains. The TCA cycle may function as the ultimate metabolic pathway for the three major nutrients in the organism [48,49]. To prevent the detrimental accumulation of metabolic intermediates within the cell, causing toxicity or negative feedback effects, bacteria must further improve the operating efficiency of key enzymes in the TCA cycle and introduce previously stored substances into downstream metabolic pathways [48].

### 3.8. ATPase Activity Analysis

ATPase is a key enzyme system that catalyzes the hydrolysis of ATP and releases energy [50]. Its synergistic effect plays an important role in biochemical processes, including cell metabolism, transmembrane transport, and signal transduction [21]. Ca^2+^/Mg^2+^-ATPase maintains the steady-state environment of various enzymatic reactions and transduces stress signals by regulating the balance of Ca^2+^ and Mg^2+^ concentrations in bacteria. Na^+^/K^+^-ATPase provides the energy for the transmembrane transport of nutrients by maintaining an ion gradient with high intracellular K^+^ and extracellular Na^+^ [50]. Figure 3E illustrates the changes in ATPase activity of each strain at different growth stages. For Ca^2+^/Mg^2+^-ATPase, the two mutant strains maintained higher enzyme activity in both the logarithmic and stationary phases, which may facilitate calcium-dependent enzymatic reactions [50]. In addition, according to the research of Bouillot et al. [51], abnormal Ca^2+^ concentration can also cause premature bacterial apoptosis. Therefore, a constant equilibrium ion concentration signifies that the strain’s activity is effectively regulated. The UV-LA strain has enzyme activity that is superior to that of the UV strain, signifying its enhanced stress resistance and a stronger ability to maintain intracellular homeostasis [21,51].

For Na^+^/K^+^-ATPase, the activity of each strain during the logarithmic growth phase was significantly higher than that in the stationary phase. Bacteria may require a high transmembrane ion gradient during the rapid proliferation stage to drive the transmembrane transport of nutrients, such as amino acids and sugars, to meet their strong anabolic needs [52]. After entering the stationary phase, due to a slowdown in basal metabolic rate, the cell’s demand for transmembrane substance transport decreases, leading to adaptive downregulation of enzyme activity [53]. The Na^+^/K^+^-ATPase activities of the two mutant strains were significantly increased, indicating that mutagenesis increased their substrate uptake and transmembrane transport efficiency, providing sufficient material support for their more vigorous growth and development. Importantly, compared with the UV strain, the UV-LA strain exhibits a smaller decline in enzyme activity from the logarithmic phase to the stationary phase, indicating that the strain still maintains physiological homeostasis during the late metabolic phase, allowing it to continue to maintain efficient transmembrane transport driving force and provide key energy guarantee for the continuous accumulation of metabolites [54].

### 3.9. Bacterial Sequencing Analysis

Whole-genome resequencing was performed on the wild strain, UV strain, and UV-LA strain, and the genetic changes occurring in the genome were analyzed. First, the genomic sequences of the UV strain and UV-LA strain were compared with those of the wild strain. Subsequently, the genetic differences between the UV and UV-LA strains were analyzed. The aim was to reveal, at the molecular level, the factors responsible for the improved phenotypes of the mutant strains relative to the wild strain, as well as the genetic basis underlying the phenotypic differences between the two mutants. The single-nucleotide polymorphism (SNP) and insertion/deletion (InDel) results identified in the two mutant strains relative to the wild strain are summarized in Table 1. A total of eight mutation sites were detected in the two mutants. Among them, six variants were identified in the UV strain, whereas only two variants were detected in the UV-LA strain. The smaller number of mutations observed in the UV-LA strain suggests that He-Ne laser treatment may have alleviated or repaired some of the UV-induced genetic damage.

In the UV strain, the most significant mutation occurred in the *yisP* gene, where a multi-base insertion or deletion caused a frameshift mutation that likely disrupted the biological activity of the encoded YisP protein. YisP is a prenyl diphosphate phosphatase involved in the non-isoprenoid pathway and/or sesquiterpene biosynthesis, and it plays an important role in regulating biofilm formation and cell surface hydrophobicity [55]. A change in YisP activity may alter cell membrane structure and permeability, thereby facilitating nutrient uptake while simultaneously promoting extracellular secretion of protease [56]. However, impairment of this enzyme may also increase bacterial sensitivity to environmental stresses such as osmotic fluctuations. This may explain why the UV strain exhibited high protease activity but lower genetic stability than the UV-LA strain during subsequent passages. Another mutation identified in the UV strain was a T to A transversion in the *iolB* gene, which may affect the activity of the encoded inositol dehydrogenase. This enzyme participates in the inositol catabolic pathway, which is generally regarded as a secondary carbon utilization pathway [57]. A change in this pathway may trigger compensatory metabolic responses that redirect carbon flux toward primary glucose metabolism, particularly glycolysis catalyzed by phosphofructokinase (PFK). This interpretation is consistent with the elevated PFK activity observed in the UV strain during previous physiological analyses.

In the UV-LA strain, a C-to-G transversion was detected in the *malP_2* gene. The encoded maltodextrin phosphorylase is an important component of bacterial carbohydrate metabolism and directly links the degradation of complex carbon sources with the glycolytic pathway [58,59]. The mutation at this locus may enhance the transfer of carbon substrates into central metabolic pathways. Compared with the disruptive mutations observed in the UV strain, the mutation in *malP_2* appears to represent a more favorable metabolic optimization. Enhanced carbohydrate utilization may increase ATP generation, thereby supporting higher physiological activity and metabolic performance in the UV-LA strain. In addition, both mutant strains carried a base transversion at *ctg00017_04167*. However, the biological function of this site has not been reported yet. Based on the physiological and proteomic results obtained in this study, it is speculated that this mutation may contribute to optimizing carbon flux distribution within central metabolism, thereby enhancing substrate uptake efficiency.

The structural variations (SV) identified in the two mutant strains are presented in Table 2. A large deletion spanning the *yisP* site was detected in the UV strain, which is consistent with the physiological effects discussed above. In contrast, the UV-LA strain contained an insertion event on ctg00013. Although the specific biological function of this site has not yet been characterized, Gene Ontology annotation indicated enrichment in ATP binding (GO:0005524) and ligase activity (GO:0016874). These functions are generally associated with ATP-dependent biosynthetic and metabolic processes [60,61]. Therefore, the insertion mutation may influence cellular energy utilization or macromolecular synthesis. However, the precise physiological consequences of this mutation remain unclear and require further functional validation.

### 3.10. Proteomic Analysis

#### 3.10.1. Differential Expression Protein Analysis

**Figure 4:**
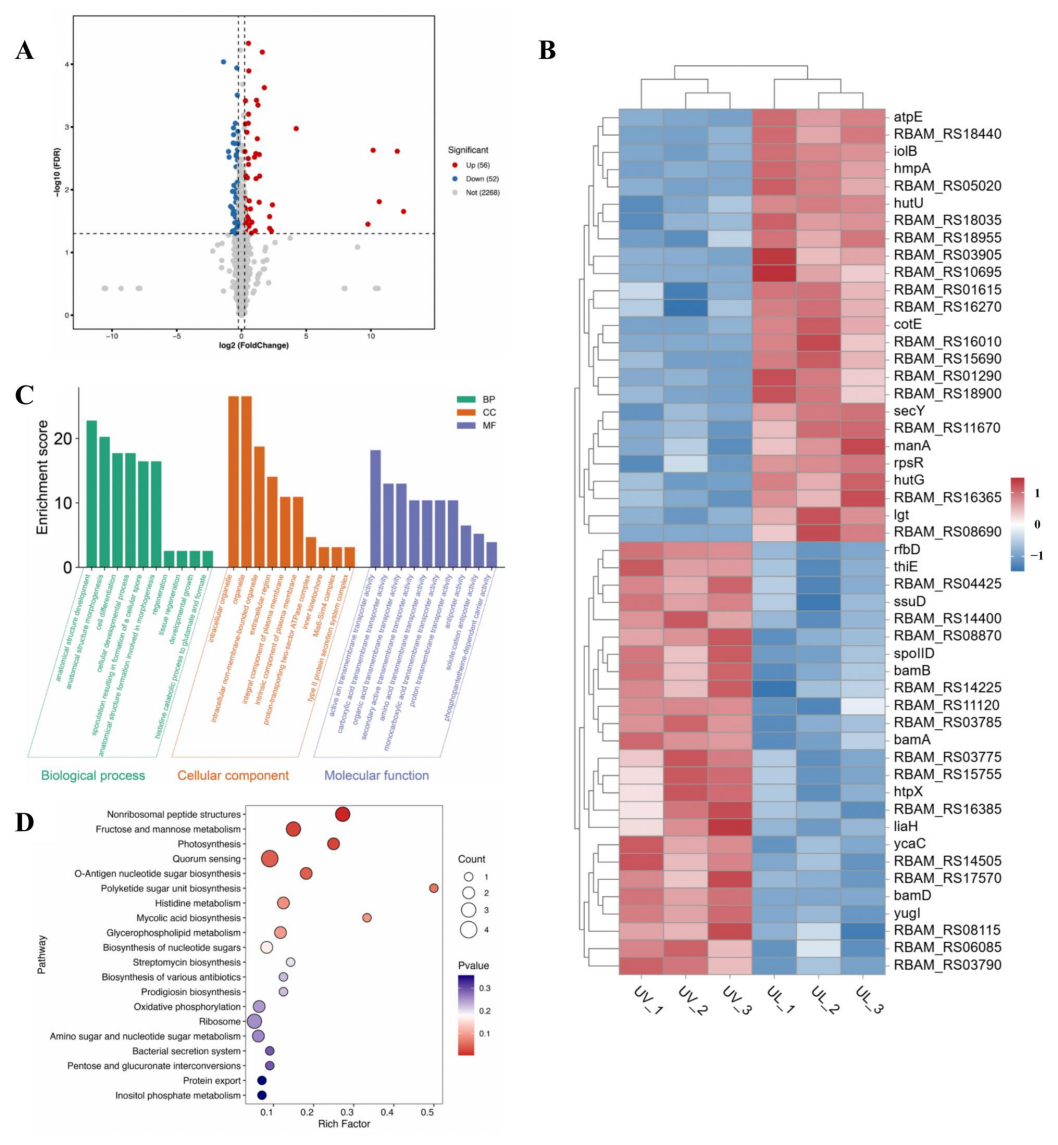
(**A**) Number of differential proteins between UV strain and UV-LA strain. (**B**) Heat map of differential proteins between UV strain and UV-LA strain. (**C**) GO enrichment analysis of differential proteins between UV strain and UV-LA strain. (**D**) KEGG pathway analysis of differential proteins between UV strain and UV-LA strain.

The differential expression at the protein level between the UV strain and the UV-LA strain is shown in Figure 4A. A total of 108 proteins were significantly different between the two groups, including 56 upregulated and 52 downregulated proteins. Most proteins are concentrated in the region where log2FC is close to 0, indicating that overall expression changes between the two groups are relatively limited. Nevertheless, certain proteins exhibit statistically significant differences, suggesting that He-Ne laser treatment may have a more pronounced regulatory effect on specific functional proteins.

#### 3.10.2. Cluster Analysis

The differential proteins of the UV and UV-LA strains were analyzed, and the results are shown in Figure 4B. Some key proteins are shown in Table 3. Among the upregulated proteins, the ATP synthase c subunit encoded by *atpE* and the 30S ribosomal protein S18 encoded by *rpsR* were significantly increased. These changes indicate that the UV-LA strain may enhance the energy flow efficiency and protein synthesis capacity, potentially providing sufficient energy and translation basis for large-scale expression of proteases [62,63]. Meanwhile, the expression levels of key proteins involved in carbon and nitrogen source utilization (*manA*, *hutU*, *hutG* and *iolB*) increased, which may promote the strain’s metabolic utilization of substrates such as mannose and histidine [57]. Notably, the *secY*-encoded transporter was upregulated. As a core membrane component of the Sec protein transport system, secY mediates the transmembrane transport of proteins. Its increased expression may enhance extracellular protein secretion, thereby partially alleviating the metabolic pressure induced by intracellular protein accumulation under high-production conditions [64].

On the other hand, the UV-LA strain may have achieved a more balanced metabolic state through the downregulation of certain non-essential proteins. The proteins encoded by *bamN*, *htpx*, and *liaH*, which are involved in outer membrane protein assembly and the cellular stress response, were downregulated, suggesting that the repair effects of the He-Ne laser treatment may reduce UV-induced damage to the bacterial structure. The expression levels of proteins encoded by *rfbD*, *thiE*, *ssuD* and *ycaC*, which are involved in the synthesis of minor metabolites and specific nutrient uptake, are reduced, indicating that cells may inhibit some non-critical metabolic pathways [65,66]. Downregulation of *spollD* and *yugl*, which encode developmental regulatory proteins, may help divert limited carbon, nitrogen, and ATP resources toward the synthesis of extracellular proteases [67]. In general, these proteomic changes suggest that He-Ne laser treatment may influence cellular resource allocation and metabolic regulation, which could be associated with the enhanced protease-producing phenotype observed in the UV-LA strain.

#### 3.10.3. GO Functional Annotation and Enrichment Analysis

To elucidate the potential biological functions of the differentially expressed proteins, GO functional enrichment analysis was performed on the proteins identified in the UV vs. UV-LA comparison group (Figure 4C). At the biological process (BP) level, differential proteins were significantly enriched in anatomical structure development (GO:0048856), anatomical structure morphogenesis (GO:0009653), cellular developmental process (GO:0048869), cell differentiation (GO:0030154), sporulation resulting in formation of a cellular spore (GO:0030435), and tissue development and morphogenesis. These results demonstrate that He-Ne laser treatment may stimulate repair mechanisms in response to UV-induced damage, which will also help to facilitate the recovery of cell development. Such changes could help maintain cellular proliferation and metabolic activity, thereby providing a more stable physiological basis for continued protease synthesis. In addition, certain processes associated with amino acid metabolism, including the histidine catabolic process to glutamate and formamide (GO:0019556), exhibit a notable degree of enrichment. This indicates that the He-Ne laser may enhance the availability of intermediate products for central metabolism by regulating the amino acid decomposition and nitrogen source utilization pathways, potentially providing a restructured carbon skeleton and nitrogen source support for subsequent protein synthesis [68]. At the cellular component (CC) level, the differential proteins were mainly located in intracellular organelles (GO:0043229), intrinsic component of membrane (GO:0031224), integral component of plasma membrane (GO:0005887), protein secretion system complex (GO:0030256), and other structural regions, indicating that these changes may involve the regulation of cell membrane structure and secretion apparatus. The optimization of secretion system functions may help promote the more efficient transport of intracellularly synthesized proteases to the outside of the cell, thereby reducing the metabolic pressure caused by intracellular protein accumulation [64]. At the molecular function (MF) level, the enriched items mainly involve active transmembrane transporter activity (GO:0022804), carboxylic acid transmembrane transporter activity (GO:0046943), and a variety of small-molecule substrate-specific transport functions. Changes in these transport-related functions may enhance the mutant strain’s ability to absorb nutrients from the culture medium and promote the transmembrane transport of metabolic intermediates and secreted proteins, thus improving the metabolic efficiency and protein secretion capacity. Overall, He-Ne laser treatment may be associated with coordinated changes in cellular repair, amino acid metabolism, membrane transport, and secretion-related processes. These changes could contribute to a more favorable physiological state for protease synthesis and secretion, thereby partially explaining the enhanced protease production observed in the UV-LA strain.

#### 3.10.4. KEGG Pathway Analysis

KEGG pathway enrichment analysis was conducted to further investigate the metabolic and signaling pathways associated with differentially expressed proteins in the comparison group, with the results presented in Figure 4D. The analysis revealed that these proteins are significantly enriched in multiple pathways related to fundamental metabolic regulation and cellular physiological processes. At the basic metabolic level, pathways such as fructose and mannose metabolism, as well as amino sugar and nucleotide sugar metabolism, are notably enriched, indicating that He-Ne laser treatment may be associated with changes in carbon metabolism and could provide additional carbon skeletons and reducing equivalents for protease synthesis [68]. Enhancing glycerophospholipid metabolism may contribute to promoting the repair and remodeling of biological membrane structures, thereby providing a stable physical environment for membrane-bound proteins and secretory channels. The activation of amino acid metabolic pathways, such as histidine metabolism, may help to provide sufficient precursor amino acids for protease synthesis [69]. Notably, the significant enrichment of protein exports and the bacterial secretion system indicates that the secretion ability may be enhanced, thereby increasing the transport rate of extracellular enzymes. Moreover, the enrichment of the oxidative phosphorylation pathway may indicate changes in energy metabolism, potentially supporting energy-demanding processes such as protein translation and transmembrane transport. Furthermore, in terms of group behavior regulation, the enrichment of quorum-sensing pathways indicates that the He-Ne laser may induce bacterial colonies to collaboratively upregulate protease gene expression during the later stages of fermentation by optimizing intercellular signaling. In summary, UV-LA treatment may help restore strain vitality by repairing the damaged metabolic network and help promote efficient protease secretion by integrating energy supply, substrate turnover, and secretion systems.

## 4. Conclusions

In this study, the repair effect of the He-Ne laser was innovatively combined with the damaging effect of UV irradiation to establish a sequential UV-LA mutagenesis strategy. Subsequently, a comparative study was conducted using UV and UV-LA treatments to induce mutations in *Bacillus velezensis* with the aim of increasing protease production. The findings indicate that, in contrast to UV treatment, UV-LA treatment not only significantly enhances the positive mutation rate but also facilitates the generation of superior mutant strains with greater increases in protease activity. Further analysis found that the physiological activity and the activities of various key metabolic enzymes of the UV-LA mutant strain were significantly higher than those of the UV mutant strain. In terms of ion transport-related ATPase activity, the UV-LA strain was superior to the UV strain in transport efficiency, confirming the overall optimization of cell membrane function by the He-Ne laser-mediated repair effect. Proteomic analysis identified 108 differentially expressed proteins between the UV strain and the UV-LA strain (56 upregulated and 52 downregulated), whose functions mainly involved bacterial energy metabolism, translation, and the secretion system. In general, UV-LA treatment may promote cell structural repair, regulate amino acid metabolism, optimize transmembrane transport and secretion processes, and re-coordinate the cellular metabolic network, mainly through the He-Ne laser’s light effects. This promotes the allocation of additional metabolic resources toward protease synthesis and secretion, ultimately improving the strain’s protease production capacity. This research is expected to introduce an innovative and effective mutagenesis method for microbial breeding and to offer new ideas for expanding the application of UV mutagenesis technology.

## Figures and Tables

**Table 1:** Statistics of SNP/InDel variants.

Sample	REF	ALT	Type	Gene
UV	G	GTGCCC	InDel	*yisP*
UV	G	GTTCAGAAAGTCTTGTGCCGCCTGTT	InDel	*yisP*
UV	A	ATCTCCCAAGTTCCTGCACATCAAG	InDel	*yisP*
UV	T	A	Transversion	*iolB*
UV	G	A	Transition	ctg00009_03167
UV	C	G	Transversion	ctg00017_04167
UV-LA	C	T	Transition	*malP_2*
UV-LA	C	G	Transversion	ctg00017_04167

**Table 2:** SV statistics.

Sample	Start	End	Type	Size	Gene
UV	437353	437548	Deletion	196	*yisP*
UV-LA	119972	119976	Insertion	5	/

**Table 3:** Key differentially expressed proteins potentially associated with protease production.

Protein/Gene	Protein Description	Log2 (FC)	*p*-Value
*iolB*	5-deoxy-glucuronate isomerase	0.538	0.000046
*atpE*	F0F1 ATP synthase subunit C	1.608	0.000064
*bamD*	Bacillomycin D biosynthesis malonyl-CoA transacylase BamD	−1.388	0.000092
*rfbD*	dTDP-4-dehydrorhamnose reductase	−0.356	0.000115
*yugL*	S1 domain-containing post-transcriptional regulator GSP13	−0.326	0.000311
*secY*	Preprotein translocase subunit SecY	0.307	0.000382
*hutU*	Urocanate hydratase	0.543	0.000625
*hutG*	Formimidoylglutamase	0.528	0.000872
*ycaC*	Isochorismate family cysteine hydrolase YcaC	−0.618	0.001035
*bamA*	Bacillomycin D hybrid PKS/NRPS BamA	−0.511	0.001837
*thiE*	Thiamine phosphate synthase	−0.274	0.001838
*spollD*	Stage II sporulation protein D	−1.002	0.002464
*bamB*	Bacillomycin D non-ribosomal peptide synthetase BamB	−0.412	0.002879
*rpsR*	30S ribosomal protein S18	0.517	0.00318
*ssuD*	FMNH2-dependent alkanesulfonate monooxygenase	−0.309	0.003365
*manA*	Mannose-6-phosphate isomerase, class I	0.338	0.006018
*liaH*	Stress-responsive protein LiaH	−0.589	0.008521
*htpX*	Protease HtpX	−0.470	0.008761

## Data Availability

The original contributions presented in the study are included in the article, and further inquiries can be directed to the corresponding author.
